# Mortality patterns of SLE and the associated risk factors in Korean patients: a nationwide cohort study

**DOI:** 10.1136/lupus-2024-001361

**Published:** 2025-02-26

**Authors:** Soo-Kyung Cho, Yena Jeon, Jung-hyo Kim, Eun Jin Jang, Sun-Young Jung, Yoon-Kyoung Sung

**Affiliations:** 1 Department of Rheumatology, Hanyang University Hospital for Rheumatic Diseases, Seongdong-gu, Korea (the Republic of); 2 Hanyang University Institute for Rheumatology Research, Seongdong-gu, Korea (the Republic of); 3 Department of Statistics, Kyungpook National University, Daegu, Korea (the Republic of); 4 Department of Data Science, Andong National University, Andong, Korea (the Republic of); 5 College of Pharmacy, Chung-Ang University, Seoul, Korea (the Republic of)

**Keywords:** Systemic Lupus Erythematosus, Mortality, Risk Factors

## Abstract

**Background:**

To evaluate the mortality patterns of SLE and the associated risk factors in Koreans.

**Methods:**

Using the National Health Insurance database spanning 2008 to 2018, incident cases of SLE in patients aged 10–79 years were included. We analysed the all-cause mortality and cause-specific mortality, stratifying by sex and age. The mortality rate (MR) was calculated as the number of deaths per 100 000 person-years (PYs). The causes of death were identified by the International Classification of Diseases, 10th Revision codes during hospitalisation or emergency visit prior to death. A generalised estimating equation model was employed for risk factor analysis.

**Results:**

In total, 11 375 incident SLE cases among patients with an average age of 42.3±16.7 years were recruited (86.1% female). During 57 658 PYs, 728 deaths occurred (MR 1262.62/100 000 PYs). The MR among men (2718.86/100 000 PYs) exceeded that among women (1060.57/100 000 PYs). The leading causes of death were SLE-related conditions (381.56/100 000 PYs), cardiovascular disease (CVD) (202.92/100 000 PYs), cancer (175.17/100 000 PYs) and infection (143.95/100 000 PYs). Of the SLE-related mortality, the key risk factors were pulmonary complications, such as pulmonary alveolar haemorrhage (OR 9.93), pulmonary arterial hypertension (OR 3.77) and interstitial lung disease (OR 3.27).

**Conclusions:**

Among Korean patients with SLE, SLE-related conditions were the leading causes of mortality. However, CVD and cancer were also identified as the main causes of mortality. Furthermore, pulmonary manifestations were significantly associated with SLE-related mortality.

**WHAT IS ALREADY KNOWN ON THIS TOPIC**

- Patients with SLE have a significantly higher mortality risk compared with the general population, with notable differences observed between racial and ethnic groups.
- The leading causes of death in SLE vary, with some studies citing SLE itself, others cardiovascular disease (CVD), or infections, reflecting differences among racial and ethnic groups.
- In Asian populations, the leading causes of mortality in SLE are debated, with studies highlighting infections, CVD-related or SLE-related conditions, underscoring the need for region-specific research.

**WHAT THIS STUDY ADDS**

- SLE-related conditions are the primary causes of death in Korean patients, followed by CVD and cancer, with higher mortality risks in men and older patients.
- Pulmonary complications are significant predictors of SLE-related mortality.
- Early deaths are linked to acute SLE manifestations, while delayed deaths are associated with chronic conditions like CVD and cancer.

**HOW THIS STUDY MIGHT AFFECT RESEARCH, PRACTICE OR POLICY**

- Tailored treatment for both acute and chronic phases of SLE could improve patient outcomes.
- Early detection and management of pulmonary complications are crucial to reducing mortality in patients with SLE.
- The study provides strong evidence to guide clinical practices in SLE management to prioritise comprehensive care, especially in Asian populations.

## Introduction

 SLE is a multisystem autoimmune disease characterised by various clinical manifestations affecting diverse organ systems.^1^ Previous studies have explored the racial and ethnic differences in the clinical presentation of SLE and the development of severe disease manifestations following diagnosis.^2 3^ A recent US epidemiological study of the California Lupus Surveillance Project (CLSP) demonstrated that patients of African-American, Asian/Pacific Islander and Hispanic ethnicity are at a higher risk of developing severe manifestations such as lupus nephritis (LN), thrombocytopenia and antiphospholipid antibody syndrome earlier than patients who identified as non-Hispanic and white.^4^

Importantly, mortality among patients with SLE has been reported to be almost four times higher than expected in those of Asian and Hispanic/Latino ethnicity, particularly among Hispanic/Latina women.^5^ In the same study, the standardised mortality ratio (SMR) of observed-to-expected deaths among persons with SLE within each racial/ethnic group was 2.3 (white), 2.0 (black), 3.8 (Asian) and 3.9 (Hispanic/Latino), respectively.^5^ Conversely, another study of Medicaid recipients showed that mortality among patients with SLE who were of Asian and Hispanic ethnicity was lower than that among those who identified as black, white or Native Americans.^6^ These discrepancies indicate that mortality cannot be explained solely by race or ethnicity. Various complex factors, such as socioeconomic status (eg, public and private health insurance and medical accessibility), drug usage and early intervention after SLE diagnosis may also be of significance.^7 8^ Additionally, SLE activity and the associated organ damage can obviously contribute to death.^9 10^ These causes of death among patients with SLE also differed between race and ethnicity. Among black people, SLE-related conditions (39%) and infection (36%) were the leading causes of death. In contrast, among white people, SLE-related conditions (44%) and cardiovascular disease (CVD) (30%) were predominant.^11^ However, recent findings from CLSP indicated that CVD has emerged as the leading cause of death across all groups, with no significant racial or ethnic differences.^12^ To accurately understand the mortality causes of patients with SLE and the associated risk factors, studies of patients with early disease and of a single race are important, especially for examining the impact of comorbidities and medications.

This study aimed to assess the all-cause and cause-specific death and mortality patterns in Koreans with SLE, stratifying by sex and age. We also investigated the potential impact of medications and comorbidities on all-cause mortality and SLE-related mortality.

## Materials and methods

### Data source and study population

We conducted a nationwide retrospective study using the Korean National Health Insurance Service (NHIS) database, which extended its coverage to approximately 97% of the population in Korea. The NHIS database houses a wealth of health-related information including demographic data, medical claims and prescription records.^13 14^

**Figure 1:**
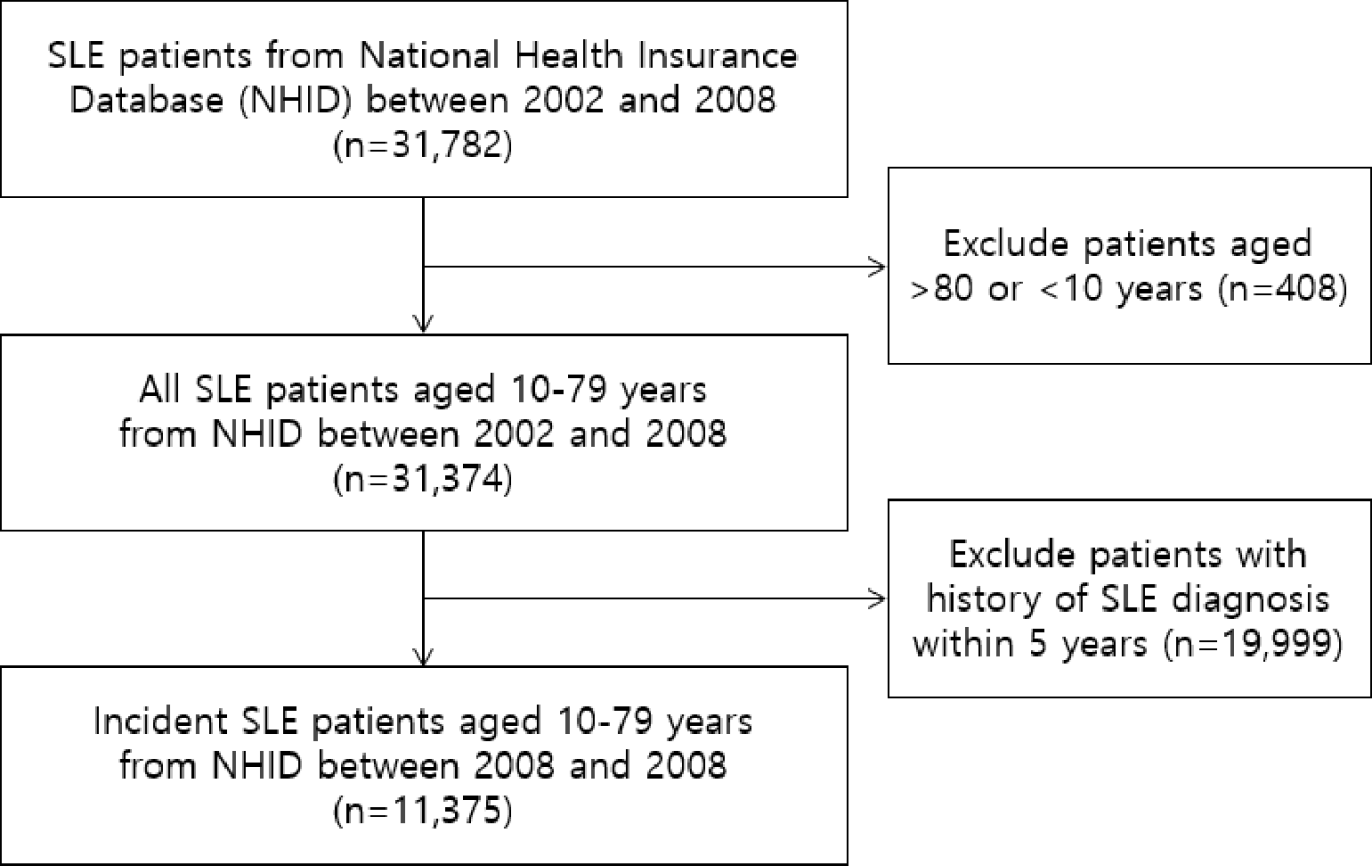
Study population selection flow.

The patients were identified from the NHIS database between 1 January 2008 and 31 December 2018. The patients’ SLE diagnosis was identified by both the International Classification of Diseases, 10th Revision (ICD-10) code (M32.0) and the rare intractable disease code (V136). The included patients also met the criteria for SLE based on the 1997 update of the 1982 American College of Rheumatology revised criteria.^15^ To ascertain the cohort of SLE incident cases, we excluded patients who had a history of SLE within the 5 years prior to the index date. Patients aged between 10 years and 79 years were enrolled (figure 1).

### Study design

The index date was defined as the date of the first SLE diagnosis. The follow-up period was defined as the time from the index date to death or the end of the study, whichever came first. Age, gender, payer type and income categories at index date were also assessed. Several chronic diseases, such as hypertension, diabetes mellitus, hyperlipidaemia, chronic kidney disease, CVD and cancer were categorised with comorbidities, while antiphospholipid antibody syndrome, avascular necrosis, interstitial lung disease, pulmonary artery hypertension, pulmonary alveolar haemorrhage and LN were categorised with SLE-related manifestations. Congestive heart failure and opportunistic infection were categorised with SLE-related complications. Comorbidities were assessed using the Charlson Comorbidity Index based on data from the year prior to the index date.^16^ SLE-related manifestations and complications were assessed during the observation period. Immunosuppressive agents included azathioprine, cyclosporine, mycophenolate mofetil, leflunomide and methotrexate. The analysis included medications that were used for more than 30 days within a year (inclusive of the index date). Additionally, intravenous cyclophosphamide or glucocorticoid injections were included if these were administered more than once, as these treatments have potential long-term effects on disease outcomes. SLE features and complications were identified throughout the entire follow-up period using diagnostic codes (ICD-10), treatment records and medication prescriptions from the Korean NHIS database.

All-cause and cause-specific deaths during the follow-up period were assessed. Cause-specific death analyses, stratified by sex and age, were also conducted. Furthermore, we evaluated the risk factors of all-cause mortality and SLE-related mortality. Additionally, we compared the leading causes of death in the early period (within 1 year of diagnosing SLE) to those in the delayed period (after 1 year of diagnosing SLE).

### Outcomes

Specific causes of death were identified using major diagnostic ICD codes from claims associated with hospitalisation or emergency visits within 6 months prior to death.^17^ These causes were categorised into several groups: SLE-related conditions, cancer, infections, cardiovascular diseases, renal disorders, other rheumatic diseases, respiratory illnesses, liver diseases, haematological disorders, mental disorders, musculoskeletal diseases, gastrointestinal conditions, injuries, accidents and suicides, endocrine disorders, sudden deaths, and unknown causes. The category ‘SLE-related condition’ was defined using the ICD-10 code M32.0 (SLE), and applied when no specific organ involvement was documented as the primary cause of death. For cases with organ-specific causes, the corresponding ICD-10 codes (eg, N18 for chronic kidney disease, J81 for pulmonary oedema or haemorrhage) were assigned. The category ‘unknown’ indicates cases where there were no hospital admissions or emergency visits within 6 months prior to death, suggesting that the death may not have occurred in a healthcare facility.

### Statistical analysis

Data are presented as the frequency (%) or mean±SD. We calculated the mortality rate (MR) per 100 000 person-years (PYs) of all-cause death and cause-specific death by dividing the number of events by the observational period and stratified by sex and age. Kaplan-Meier graphs were constructed for all patients and by sex, and the difference in survival probabilities by gender was compared using the log-rank test. To assess the influence of various risk factors on all-cause mortality and SLE-related mortality, the generalised estimating equation (GEE) method was applied. The GEE method allows for the incorporation of time-dependent covariates, which are factors that may change over the course of the study, such as comorbidities, SLE-related manifestations, complications and medications. Variables for comorbidities, medications and mortality were created at 1-year intervals, and a GEE model was fitted. The ORs and 95% CIs are presented. The leading causes of death in the early period were compared with those of the late period and presented as the frequency of events. The risk factors for SLE-related early deaths were assessed using logistic regression. All analyses were performed using SAS V.9.4 (SAS Institute, Cary, North Carolina, USA). A value of p<0.05 was considered statistically significant.

## Results

### Baseline characteristics

In total, 11 375 patients with newly diagnosed SLE were identified from the Korean NHIS database during the study period. The average age of the incident patients was 42.3±16.7 years, with 86.1% being female. Hypertension (20.5%), diabetes mellitus (4.9%) and hyperlipidaemia (11.6%) were prevalent among patients with SLE. The most common SLE-related comorbidity during the observational period was opportunistic infections (19.8%), followed by interstitial lung disease (4.9%) and antiphospholipid antibody syndrome (4.7%). Among the incident SLE cases, 70.3% received oral glucocorticoids and 68.8% were treated with hydroxychloroquine (table 1).

**Table 1:** Baseline characteristics of patients with incident SLE

Variables	Patients with incident SLE(n=11 375)	Male(n=1576)	Female(n=9799)
Age, years	42.3±16.7	44.4±18.7	41.9±16.4
10–19	1026 (9.0)	165 (10.5)	861 (8.8)
20–29	1939 (17.0)	270 (17.1)	1669 (17.0)
30–39	2217 (19.5)	236 (15.0)	1981 (20.2)
40–49	2311 (20.3)	233 (14.8)	2078 (21.2)
50–59	1913 (16.8)	259 (16.4)	1654 (16.9)
60–69	1180 (10.4)	250 (15.9)	930 (9.5)
70–79	789 (6.9)	163 (10.3)	626 (6.4)
Payer type			
National health insurance	10 061 (88.4)	1296 (82.2)	8765 (89.4)
Medical aid	1314 (11.6)	280 (17.8)	1034 (10.6)
Charlson Comorbidity Index	2.6±1.7	3.0±2.0	3.0±1.7
Comorbidities*			
Hypertension	2335 (20.5)	469 (29.8)	1866 (19.0)
Diabetes mellitus	554 (4.9)	137 (8.7)	407 (4.2)
Hyperlipidaemia	1324 (11.6)	261 (16.6)	1063 (10.8)
Chronic kidney disease	407 (3.6)	100 (6.3)	307 (3.1)
Cardiovascular disease	394 (3.5)	114 (7.2)	280 (2.9)
Cancer	513 (4.5)	75 (4.8)	438 (4.5)
SLE-related manifestations†			
Antiphospholipid antibody syndrome	535 (4.7)	85 (5.4)	450 (4.6)
Avascular necrosis	398 (3.5)	83 (5.3)	315 (3.2)
Interstitial lung disease	561 (4.9)	97 (6.2)	464 (4.7)
Pulmonary artery hypertension	155 (1.4)	18 (1.1)	137 (1.4)
Pulmonary alveolar haemorrhage	69 (0.6)	12 (0.8)	57 (0.6)
Lupus nephritis	2298 (20.2)	345 (21.9)	1953 (19.9)
SLE-related complications†			
Congestive heart failure	1295 (11.4)	246 (15.6)	1049 (10.7)
Opportunistic infection	2254 (19.8)	282 (17.9)	1972 (20.1)
Medication‡			
Glucocorticoid (oral)	7993 (70.3)	1069 (67.8)	6924 (70.7)
Glucocorticoid (intravenous)	5654 (49.7)	828 (52.5)	4826 (49.2)
Hydroxychloroquine	7827 (68.8)	866 (54.9)	6961 (71.0)
Non-steroidal anti-inflammatory drug	4738 (41.7)	577 (36.6)	4161 (42.5)
Immunosuppressive agent	3789 (33.3)	559 (35.5)	3230 (33.0)
Cyclophosphamide (intravenous)	966 (8.5)	174 (11.0)	792 (8.1)

Numerical quantitative data were are presented by ‘mean±SD’ and categorical data were are presented by ‘frequency (%)’”.Comorbidities were assessed within a year before the index date. SLE-related manifestations and complications were assessed during observational periods. Medication use for more than within a year including the index date.

* Comorbidities were assessed within a year before the index date.

† SLE-related manifestations and complications were assessed during observational periods.

‡ Medication use for more than 30 days within a year including the index date.

SLEsystemic lupus erythematosus

### All-cause and cause-specific MRs

Among the patients with incident SLE (n=11 375), there were 728 deaths over 57 658 PYs, resulting in an all-cause MR of 1262.62 per 100 000 PYs. The average follow-up duration was 4.70 years. The most common cause of death was SLE-related condition (30.2%), followed by CVD (16.1%), cancer (13.9%) and infection (11.4%). Men had a significantly higher all-cause MR (2718.86/100 000 PYs) compared with women (1060.57/100 000 PYs) (p<0.001; online supplemental figure). In contrast, the major causes of death did not differ significantly between women and men (table 2).

**Table 2:** All-cause mortality and cause-specific mortality in all patients with SLE and sex-stratified patients with SLE

	All patients (n=11 375)			Female (n=9799)			Male (n=1576)		
	Observational period (PYs)	No. of events	MR per 100 000 PYs (95% CI)	Observational period (PYs)	No. of events	MR per 100 000 PYs (95% CI)	Observational period (PYs)	No. of events	MR per 100 000 PYs (95% CI)
All cause	57 658	728	1262.62(1170.90 to 1354.34)	50 633	537	1060.57(970.87 to 1150.28)	7025	191	2718.86(2333.27 to 3104.45)
SLE-related condition	57 658	220	381.56(331.14 to 431.98)	50 633	180	353.50(303.56 to 407.43)	7025	40	569.40(392.94 to 745.85)
Cardiovascular	57 658	117	202.92(166.15 to 239.69)	50 633	78	154.05(119.86 to 188.24)	7025	39	555.16(380.92 to 729.40)
Cancer	57 658	101	175.17(141.01 to 209.33)	50 633	65	128.37(97.17 to 159.58)	7025	36	512.46(345.05 to 679.86)
Infection	57 658	83	143.95(112.98 to 174.92)	50 633	57	112.57(83.35 to 141.80)	7025	26	370.11(227.84 to 512.37)
Renal	57 658	33	57.23(37.71 to 76.76)	50 633	29	57.27(36.43 to 98.12)	7025	4	56.94(1.14 to 112.74)
Rheumatoid	57 658	17	29.48(15.47 to 43.50)	50 633	14	27.65(13.17 to 42.13)	7025	3	42.70(−5.62 to 91.03)
Respiratory	57 658	15	26.02(12.85 to 39.18)	50 633	10	19.75(7.51 to 31.99)	7025	5	71.17(8.79 to 133.56)
Liver	57 658	14	24.28(11.56 to 37.00)	50 633	14	27.65(13.17 to 42.13)	7025	0	–
Haematological	57 658	12	20.81(9.04 to 32.59)	50 633	10	19.75(7.51 to 31.99)	7025	2	28.47(−10.99 to 67.93)
Sudden death	57 658	9	15.61(5.41 to 25.81)	50 633	9	17.77(6.16 to 29.39)	7025	0	–
Gastrointestinal	57 658	9	15.61(5.41 to 25.81)	50 633	9	17.77(6.16 to 29.39)	7025	0	–
Mental	57 658	8	13.87(4.26 to 23.49)	50 633	5	9.87(1.22 to 18.53)	7025	3	42.70(−5.62 to 91.03)
Injury	57 658	5	8.67(1.07 to 16.27)	50 633	4	7.90(0.16 to 15.64)	7025	1	14.23(−13.67 to 42.14)
Musculoskeletal	57 658	5	8.67(1.07 to 16.27)	50 633	2	3.95(−1.52 to 9.42)	7025	3	42.70(−5.62 to 91.03)
Accident/suicide	57 658	3	5.20(−0.68 to 11.09)	50 633	2	3.95(−1.52 to 9.42)	7025	1	14.23(−13.67 to 42.14)
Endocrine	57 658	3	5.20(−0.68 to 11.09)	50 633	2	3.95(−1.52 to 9.42)	7025	1	14.23(−13.67 to 42.14)
Unknown	57 658	22	38.16(22.21 to 54.10)	50 633	15	29.62(14.63 to 44.62)	7025	7	99.64(25.83 to 173.46)
Uncategorised	57 658	52	90.19(65.67 to 114.70)	50 633	32	63.20(41.30 to 85.10)	7025	20	284.70(159.92 to 409.47)

MR, mortality ratePY, person-year

The age-stratified MR showed an increasing trend with age. The highest MR was observed in elderly patients aged 70–79 years (7252.06 per 100 000 PYs). In younger age groups, particularly those under 29 years of age, SLE-related conditions were the most common cause of death. Moreover, the MR for SLE-related deaths was 381.68 per 100 000 PYs among patients aged 10–19 years. As patients transitioned to adulthood (30–39 years of age), cancer-related mortality increased (MR reaching 47.20 per 100 000 PYs). Notably, a significant rise in CVD-related deaths was detected in patients aged 40–49 years (MR of 129.81 per 100 000 PYs). This trend continued upwards, with a marked increase observed in patients aged 50–59 years, where the MR for CVD-related deaths rose to 306.71 per 100 000 PYs (table 3).

**Table 3:** Age-stratified MR among patients with SLE

Age group, years	MR per 100 000 PYs (95% CI)						
	10–19	20–29	30–39	40–49	50–59	60–69	70–79
All-cause	520.47(334.22 to 706.72)	630.47(479.50 to 781.44)	590.04(456.50 to 723.58)	754.50(601.16 to 907.85)	1456.90(1209.29 to 1704.50)	3186.81(2668.10 to 3705.53)	7252.06(6207.02 to 8297.09)
SLE-related condition	381.68(222.19 to 541.17)	366.99(251.81 to 482.17)	283.22(190.70 to 375.74)	227.16(143.02 to 311.30)	328.62(211.03 to 446.22)	769.23(514.38 to 1024.08)	1176.01(755.18 to 1596.84)
Cardiovascular	34.70(−13.39 to 82.79)	84.69(29.36 to 140.02)	47.20(9.43 to 84.97)	129.81(66.20 to 193.41)	306.71(193.11 to 420.32)	571.43(351.78 to 791.08)	1176.01(755.18 to 1596.84)
Cancer	34.70(−13.39 to 82.79)	0	47.20(9.43 to 84.97)	129.81(66.20 to 193.41)	240.99(140.29 to 341.69)	483.52(281.47 to 685.57)	1293.61(852.24 to 1734.98)
Infection	0	28.23(−3.72 to 60.18)	31.47(0.63 to 62.31)	73.02(25.31 to 120.72)	131.45(57.07 to 205.82)	483.52(281.47 to 685.57)	1293.61(852.24 to 1734.98)
Renal	0	37.64(0.75 to 74.53)	31.47(0.63 to 62.31)	24.34(−3.20 to 51.88)	76.68(19.87 to 133.48)	153.85(39.88 to 267.82)	313.60(96.29 to 530.92)

MR, mortality rate; PY, person-years

### Risk factors for all-cause and SLE-related deaths

For all-cause mortality, significant risk factors included advanced age (OR 1.04, 95% CI 1.03 to 1.05), chronic kidney disease (OR 2.66, 95% CI 1.75 to 2.91), CVD (OR 2.27, 95% CI 1.78 to 2.89) and cancer (OR 2.65, 95% CI 2.12 to 3.32). Other factors are presented in table 4.

**Table 4:** Risk factors for mortality in patients with SLE (n=11 375)

Variables	All-cause death (n=728)				SLE-related death (n=220)			
	Univariable OR(95% CI)	P value	Multivariable OR(95% CI)	P value	Univariable OR(95% CI)	P value	Multivariable OR(95% CI)	P value
Age	1.05 (1.04 to 1.06)	<0.001	1.04 (1.03, 1.05)	<0.001	1.01 (1.00 to 1.02)	0.057	1.00 (0.99 to 1.01)	0.798
Female	0.38 (0.32 to 0.45)	<0.001	0.52 (0.43, 0.64)	<0.001	0.62 (0.44 to 0.88)	0.007	0.80 (0.53 to 1.19)	0.268
Medical aid	1.25 (0.99 to 1.58)	0.056	0.69 (0.53, 0.90)	0.006	0.32 (0.15 to 0.68)	0.003	0.21 (0.10 to 0.44)	<0.001
Comorbidities†								
Hypertension	2.33 (2.01 to 2.71)	<0.001	1.02 (0.84 to 1.24)	0.851	2.12 (1.62 to 2.76)	<0.001	1.14 (0.81 to 1.60)	0.449
Diabetes mellitus	2.66 (2.13 to 3.32)	<0.001	0.99 (0.74 to 1.33)	0.940	1.74 (1.10 to 2.77)	0.019	1.03 (0.56 to 1.88)	0.934
Hyperlipidaemia	1.07 (0.89 to 1.30)	0.462	0.37 (0.29 to 0.47)	<0.001	1.05 (0.75 to 1.48)	0.462	0.54 (0.36 to 0.81)	0.003
Chronic kidney disease	5.15 (4.23 to 6.27)	<0.001	2.66 (1.75 to 2.91)	<0.001	4.70 (3.31 to 6.69)	<0.001	1.84 (1.14 to 2.96)	0.012
Cardiovascular disease	5.71 (4.89 to 6.67)	<0.001	2.27 (1.78 to 2.89)	<0.001	4.27 (3.21 to 5.67)	<0.001	1.61 (0.99 to 2.62)	0.056
Cancer	5.16 (4.26 to 6.26)	<0.001	2.65 (2.12 to 3.32)	<0.001	0.94 (0.50 to 1.78)	<0.001	0.36 (0.17 to 0.75)	<0.001
SLE-related manifestations‡								
Antiphospholipid antibody syndrome	0.99 (0.62 to 1.58)	0.958	0.80 (0.49 to 1.32)	0.389	0.71 (0.26 to 1.91)	0.500	0.54 (0.20 to 1.44)	0.218
Avascular necrosis	1.08 (0.63 to 1.84)	0.788	1.37 (0.78 to 2.42)	0.269	1.00 (0.37 to 2.69)	0.997	1.27 (0.46 to 3.47)	0.643
Interstitial lung disease	4.84 (3.77 to 6.20)	<0.001	2.75 (2.00 to 3.80)	<0.001	5.34 (3.51 to 8.14)	<0.001	3.27 (1.87 to 5.72)	<0.001
Pulmonary artery hypertension	6.82 (4.48 to 10.39)	<0.001	3.59 (1.97 to 6.54)	<0.001	8.87 (4.70 to 16.76)	<0.001	3.77 (1.54 to 9.21)	0.004
Pulmonary alveolar haemorrhage	32.24 (18.70 to 55.58)	<0.001	12.32 (5.17 to 29.34)	<0.001	54.86 (29.49 to 102.04)	<0.001	9.93 (3.81 to 25.89)	<0.001
Lupus nephritis	2.98 (2.49 to 3.55)	<0.001	1.61 (1.28 to 2.03)	<0.001	4.58 (3.44 to 6.10)	<0.001	2.15 (1.46 to 3.18)	<0.001
SLE-related complications‡								
Congestive heart failure	7.66 (6.38 to 9.20)	<0.001	2.27 (1.73 to 2.98)	<0.001	7.79 (5.69 to 10.65)	<0.001	3.06 (1.83 to 5.14)	<0.001
Opportunistic infection	2.98 (2.38 to 3.73)	<0.001	1.54 (1.17 to 2.02)	0.002	4.41 (3.11 to 6.27)	<0.001	2.10 (1.37 to 3.24)	0.001
Medication*								
Glucocorticoid (oral)	0.84 (0.72 to 0.97)	0.021	0.95 (0.78 to 1.15)	0.594	0.88 (0.68 to 1.15)	0.356	0.64 (0.44 to 0.93)	0.018
Glucocorticoid (intravenous)	5.29 (4.47 to 6.26)	<0.001	4.35 (3.62 to 5.23)	<0.001	19.34 (12.13 to 30.85)	<0.001	16.38 (10.06 to 26.66)	<0.001
Hydroxychloroquine	0.36 (0.30 to 0.42)	<0.001	0.41 (0.34 to 0.49)	<0.001	0.39 (0.30 to 0.51)	<0.001	0.39 (0.28 to 0.56)	<0.001
Non-steroidal anti-inflammatory drug	0.49 (0.41 to 0.59)	<0.001	0.33 (0.27 to 0.40)	<0.001	0.36 (0.25 to 0.52)	<0.001	0.32 (0.22 to 0.49)	<0.001
Immunosuppressive agent (oral)	1.02 (0.87 to 1.19)	0.845	0.59 (0.47 to 0.74)	<0.001	0.64 (0.46 to 0.87)	0.0048	0.35 (0.23 to 0.54)	<0.001
Cyclophosphamide (intravenous)	6.05 (4.85 to 7.54)	<0.001	3.76 (2.75 to 5.14)	<0.001	14.32 (10.58 to 19.39)	<0.001	5.51 (3.38 to 8.97)	<0.001

* Medication prescription more than 30 days within a year., Comorbidities were assessed within a year before the index date. SLE-related manifestations and complications were assessed during observational periods. Medication use for more than within a year including the index date.

† Comorbidities were assessed within a year before the index date.

‡ SLE-related manifestations and complications were assessed during observational periods.

ORodds ratioSLEsystemic lupus erythematosus

For SLE-related mortality, the effects of age and sex were not significant. Chronic kidney disease (OR 1.84, 95% CI 1.14 to 2.96) remained a risk factor. Opportunistic infections (OR 2.10, 95% CI 1.37 to 3.24), pulmonary complications including pulmonary alveolar haemorrhage (OR 9.93, 95% CI 3.81 to 25.89), pulmonary arterial hypertension (OR 3.77, 95% CI 1.54 to 9.21), interstitial lung disease (OR 3.27, 95% CI 1.54 to 6.94), congestive heart failure (OR 3.06, 95% CI 1.83 to 5.14) and LN (OR 2.15, 95% CI 1.46 to 3.18) were consistently associated with increased SLE-related mortality. Moreover, glucocorticoid and cyclophosphamide usage were significant risk factors. In contrast, the use of hydroxychloroquine, immunosuppressive agents and non-steroidal anti-inflammatory drugs (NSAIDs) all trended towards decreasing risks.

### Early deaths compared with delayed deaths

We compared the leading causes of death in the early period (within 1 year of diagnosing SLE) compared with those in the delayed period. Patients who died early often had more severe disease manifestations at diagnosis, including higher rates of LN and severe pulmonary complications, such as pulmonary alveolar haemorrhage (online supplemental table 1). The primary cause of early death was SLE-related condition, which indicated that acute SLE manifestations and severe disease presentations significantly contributed to mortality within the first year following a diagnosis. In contrast, delayed deaths were more frequently associated with chronic comorbidities, such as CVD and cancer (online supplemental table 2). Notably, the use of intravenous glucocorticoids was associated with a markedly increased risk of early death (OR 15.41, 95% CI 7.85 to 30.24). The use of intravenous glucocorticoids may be indicative of SLE severity. Other significant risk factors included congestive heart failure (OR 3.12, 95% CI 1.88 to 5.17) and interstitial lung disease (OR 2.87, 95% CI 1.45 to 5.70), indicating the negative impact of severe systemic involvement on patient outcomes. Opportunistic infections also showed a significant association (OR 1.73, 95% CI 1.01 to 2.96) (online supplemental table 3).

## Discussion

In this nationwide observational study, the all-cause MR of SLE was 1260.62 per 100 000 PYs. This rate is consistent with those reported previously (990 and 1580 deaths per 100 000 PYs based on a nationwide cohort study).^10 18^ The most common cause of death among Koreans with SLE was SLE-related conditions, while CVD and cancer have emerged as contributing factors to SLE mortality. Notably, there was a marked increase in CVD-related deaths among patients aged 40 years and over. Early deaths were primarily due to SLE-related conditions, while CVD and cancer became more prevalent causes of death later.

In Korea, patients with SLE had an increased death risk (SMR of 3.02).^3^ This was slightly lower than the SMR of 3.8 in Asian groups from a US registry (CLSP) and higher than the SMR of white (2.3) and black (2.0) individuals.^4^ In 2005, a previous single-centre Korean study demonstrated a MR of 596.0 per 100 000 PYs in women with SLE, and most deaths were attributed to SLE-related conditions.^19^ Another single-centre Korean study of SLE from 2011 found that the most common cause of death was infection (37.3%), followed by SLE-related complications (22.0%).^20^ These findings suggest that while advances in medical therapy and treatment strategies may have improved overall SLE management, the risks of infections may be problematic. This is possibly due to the use of high-dose steroids or aggressive immunosuppressive therapies. In our study, we observed a higher MR of 1060.57 per 100 000 PYs in women. This discrepancy may be influenced by study design differences. Our nationwide, population-based study included a diverse range of hospitals and clinics, potentially capturing a broader spectrum of patient management strategies and outcomes. In contrast, previous studies were conducted in specialised centres for rheumatic diseases, where patients likely receive more regular follow-up and strict monitoring. Additionally, our study focused on incident SLE cases, which may have more accurately detected early deaths. In contrast, previous studies included prevalent cases, thus potentially contributing to the differences observed in MRs.

Recent reports from CLSP indicated that CVD is the leading cause of death, followed by rheumatic disease and haematological/oncological conditions. This pattern is consistent with findings from other population-based cohort studies, highlighting the increased risk of CVD and mortality among patients with SLE compared with the general population.^7 21 22^ In contrast, a multicentre study from China reported different causes of death, identifying infections as the most common cause of death, followed by LN, haematological abnormality, neuropsychiatric lupus and interstitial pneumonia.^23^ In our study, the most common cause of death was SLE-related conditions, followed by CVD. Notably, deaths due to CVD were more frequent years after SLE diagnosis. The different causes of death may be attributed to the tendency for lower CVD rates among Asian populations compared with Western populations.^24^

Our data also revealed sex differences in mortality, with men experiencing approximately 2.7 times higher MR than women, particularly in CVD. These findings aligned with previous findings. Men with SLE tend to present with more severe disease manifestations and an increased risk of cumulative organ damage over time, which is a critical factor contributing to poorer prognosis. The combination of more aggressive disease activity and the higher likelihood of damage accrual may partly explain the observed gender disparities in MRs.^25 26^ We also analysed the characteristics of MR according to the age at onset. In patients aged under 30 years, SLE-related conditions were the predominant cause of mortality, accounting for over 50% of cases. This underscores the need for early diagnosis and aggressive treatment to mitigate the potentially fatal consequences of SLE. Among those in the 40–59 years age group, mortality patterns broadened to include SLE-related conditions, CVD, cancer and infection. Among those over 70 years of age, cancer and infection predominated, while SLE-related conditions contributed less to mortality, possibly due to lower disease activities in older patients.^27^ These results emphasised the importance of vigilant cancer screening and infection prevention measures to enhance overall survival outcomes among older adults with SLE. The vulnerability to mortality in our study population was also most pronounced in individuals aged over 60 years. These findings highlight the need for age-specific management strategies in SLE. Younger patients are prone to SLE-related complications such as LN and infections; therefore, they require aggressive disease control and early intervention to prevent mortality. In contrast, older patients tend to have a higher burden of comorbidities, such as CVD and cancer; therefore, they may benefit more from regular monitoring, cancer screening and infection prevention. These patterns underscore the importance of a tailored, holistic approach to SLE care across the lifespan. A higher MR among elderly patients with SLE than those of childbearing age was previously reported.^28^ However, another previous study demonstrated that the mortality risk in elderly patients with SLE was not significantly elevated compared with the rates observed in a non-SLE population.^29^ Taken together, these findings may reflect the influence of ageing and the associated accrual of comorbidities over the study period.^30^

The analysis of early versus delayed mortality among patients with SLE revealed significant differences in the timing and causes of death. Patients who experienced early deaths, occurring within the first year after diagnosis, had more acute SLE manifestations and severe disease presentations. These findings support that more aggressive forms of SLE, along with treatment complications due to high doses of glucocorticoids and cyclophosphamide, significantly contribute to early mortality. In contrast, delayed deaths were more often associated with chronic comorbidities, particularly CVD and cancer. The bimodal distribution of mortality in SLE was reported in 1976,^31^ with fatalities accumulating within the first year after diagnosis, mostly attributed to disease activity, and probably also treatment complications. This bimodal mortality pattern has also been confirmed in other populations.^32 33^ However, in our cohort, we found no substantial differences between the contributing factors of early deaths and those of overall mortality. These findings highlight the need to incorporate detailed patient-level clinical data to better delineate the distinctions between early and delayed mortality in future studies.

In this study, advanced age was identified as a significant risk factor for mortality in patients with SLE. Additionally, SLE-related manifestations, such as pulmonary alveolar haemorrhage, pulmonary artery hypertension and interstitial lung disease, were found to be strongly associated with increased mortality risks. Although these pulmonary conditions are relatively rare, they have a severe clinical course, thus significantly impacting the overall prognosis of patients with SLE.^34 35^ Our results concurred with the findings from previous studies that highlighted the substantial impact of pulmonary manifestations on overall SLE prognosis.^36 37^ Moreover, we found that the use of intravenous glucocorticoid or cyclophosphamide was associated with mortality. However, these results may reflect high disease activities among patients who require high-dose glucocorticoids or immunosuppressants, rather than a direct effect of the medications.^38^ Early recognition and timely management, such as immunosuppressive therapy for pulmonary alveolar haemorrhage or targeted therapy for pulmonary hypertension, are crucial for improving outcomes. Routine screening and a multidisciplinary approach may further enhance early detection and intervention, thus reducing mortality risks associated with these conditions. These findings underscore the importance of addressing life-threatening conditions including pulmonary manifestations and implementing tailored interventions across both acute and chronic phases of SLE to improve survival rates and quality of life across the SLE spectrum. By understanding these risk factors, clinicians can better tailor their therapeutic approaches to improve the survival outcomes of patients with SLE. Importantly, further research is needed to determine whether early recognition and intervention for these complications directly translate to improved long-term outcomes.

The strength of this study rests with the use of NHIS data, which provided a high degree of generalisability in Korea. The use of national data, which cover all Korean patients, avoids potential selection bias common to cohort studies. This study was also the first to demonstrate the SLE-related mortality risk of Koreans in a population-based cohort study. Moreover, we comprehensively analysed the overall and cause-specific MRs to identify the characteristic mortality patterns among incident SLE cases, including stratified analyses of the age at onset and sex. Furthermore, the use of the GEE method allowed for the incorporation of time-dependent covariates, which is particularly important in longitudinal studies, where factors like medications may change over time.

Our study also has several limitations. First, while we categorised causes of death into detailed groups, the classification of ‘SLE-related condition’ was based on the ICD-10 code M32.0, and represented deaths that were broadly attributed to SLE without specific documentation of any specific organ involvement. Second, the absence of information on disease activity limits our ability to fully assess the impact of disease severity on mortality. While we included various manifestations, medications and some socioeconomic variables, such as payer type and income, relying on diagnostic codes may have led to potential underestimation or overestimation of certain manifestations, such as interstitial lung disease interstitial lung disease (ILD) or antiphospholipid antibody syndrome (APS). Furthermore, the lack of detailed clinical and socioeconomic data such as disease activity scores or comprehensive patient-level information represents a limitation that should be addressed in future studies. Third, our study did not calculate SMR using a matched general population cohort due to the lack of access to an appropriate data set for comparison. Additionally, paediatric patients (younger than 10 years of age) were excluded from this study, as our focus was on adult-onset SLE. This exclusion may limit the generalisability of our findings.

## Conclusion

Our nationwide study reveals that the leading cause of death in Koreans with SLE is disease-related conditions, with increasing contributions from CVD and cancer. Men and those over 60 years of age face higher mortality risks, primarily from CVD. Early mortality is often due to severe SLE manifestations, while later mortality is linked to comorbidities, such as CVD and cancer. Pulmonary manifestations were significantly associated with SLE-related mortality. These findings highlight the need for comprehensive management strategies addressing both the acute and chronic aspects of SLE to improve patient outcomes.

## supplementary material

10.1136/lupus-2024-001361 online supplemental file 1

## Data Availability

Data may be obtained from a third party and are not publicly available.
