# Supplementary material for: Mortality patterns of SLE and the associated risk factors in Korean patients: a nationwide cohort study
Source: Lupus Sci Med. 2025 Feb 26;12(1):e001361. doi: 10.1136/lupus-2024-001361 (PMC11865735; doi:10.1136/lupus-2024-001361)
Supplement: online supplemental file 1 [file lupus-12-1-s001.docx]

**Supplementary Figure. Kaplan-Meire curve of overall death**

1. **Total**

**
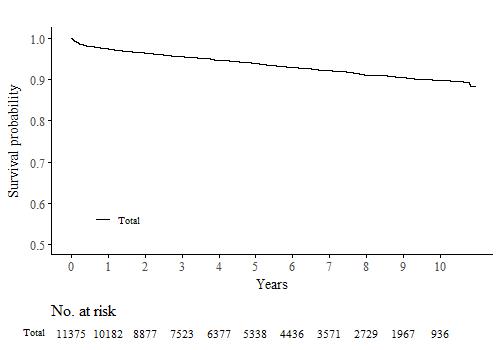
**

1. **Male vs. Female**

**
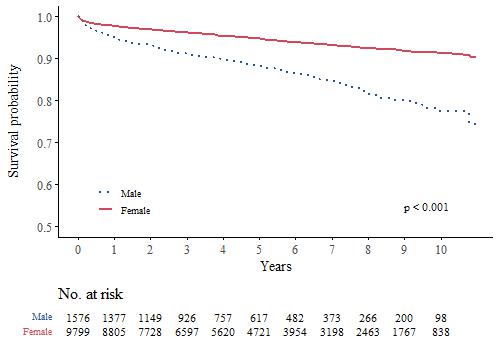
**

Survival probability

|  | 1year | 2year | 3 year | 4year | 5year | 6year | 7year | 8year | 9year | 10year |
| --- | --- | --- | --- | --- | --- | --- | --- | --- | --- | --- |
| Total | 0.974 | 0.964 | 0.955 | 0.947 | 0.939 | 0.929 | 0.921 | 0.910 | 0.904 | 0.897 |
| Male | 0.952 | 0.933 | 0.910 | 0.899 | 0.881 | 0.862 | 0.845 | 0.816 | 0.802 | 0.775 |
| Female | 0.977 | 0.969 | 0.962 | 0.954 | 0.948 | 0.939 | 0.933 | 0.924 | 0.919 | 0.914 |

**Supplementary Table 1. Baseline characteristics of patients according to the time of death**

| Variables | Early death  (n=290) | Late death  (n=438) | p-value |
| --- | --- | --- | --- |
| Age, years | 53.3 ± 19.2 | 54.4 ± 17.3 | 0.463 |
| 10-19 | 13 (4.5) | 17 (3.9) | 0.003 |
| 20-29 | 38 (13.1) | 29 (6.6) |  |
| 30-39 | 27 (9.3) | 48 (11.0) |  |
| 40-49 | 30 (10.3) | 63 (14.4) |  |
| 50-59 | 40 (13.8) | 96 (21.2) |  |
| 60-69 | 69 (23.8) | 76 (17.4) |  |
| 70-79 | 73 (25.2) | 112 (25.6) |  |
| Gender |  |  |  |
| Male | 74 (25.5) | 117 (26.7) | 0.720 |
| Female | 216 (74.5) | 321 (73.3) |  |
| Payer type |  |  |  |
| National health insurance | 263 (90.7) | 397 (90.6) | 0.982 |
| Medical aid | 27 (9.3) | 41 (9.4) |  |
| Charlson comorbidity index | 3.87 ± 2.47 | 3.39 ± 2.14 | 0.007 |
| Comorbidities^†^ |  |  |  |
| Hypertension | 132 (45.5) | 171 (39.0) | 0.083 |
| Diabetes mellitus | 36 (12.4) | 48 (11.0) | 0.548 |
| Hyperlipidaemia | 61 (21.0) | 74 (16.9) | 0.160 |
| Chronic kidney disease | 26 (9.0) | 38 (8.7) | 0.893 |
| Cardiovascular disease | 39 (13.4) | 43 (9.8) | 0.129 |
| Cancer | 24 (8.3) | 29 (6.6) | 0.400 |
| SLE-related manifestations^††^ |  |  |  |
| Antiphospholipid antibody syndrome | 7 (2.4) | 14 (3.2) | 0.537 |
| Avascular necrosis | 3 (1.0) | 7 (1.6) | 0.522 |
| Interstitial lung disease | 32 (11.0) | 51 (11.6) | 0.800 |
| Pulmonary artery hypertension | 11 (3.8) | 9 (2.1) | 0.160 |
| Pulmonary alveolar haemorrhage | 14 (4.8) | 3 (0.7) | < 0.001 |
| Lupus nephritis | 100 (34.5) | 250 (57.1) | 0.007 |
| SLE-related complications |  |  |  |
| Congestive heart failure | 82 (28.3) | 56 (12.8) | <0.0001 |
| Opportunistic infe ction | 45 (15.5) | 49 (11.2) | 0.088 |
|  |  |  |  |
| Medication^§^ |  |  |  |
| Glucocorticoid (oral) | 140 (88.3) | 350 (79.9) | <0.0001 |
| Glucocorticoid (intravenous) | 256 (88.3) | 257 (58.7) | <0.0001 |
| Hydroxychloroquine | 107 (36.9) | 268 (61.2) | <0.0001 |
| Nonsteroidal anti-inflammatory drug | 46 (15.9) | 179 (40.9) | <0.0001 |
| Immunosuppressive agent | 53 (18.3) | 164 (37.4) | <0.0001 |
| Cyclophosphamide (intravenous) | 63 (21.7) | 46 (10.5) | <0.0001 |

Numerical quantitative data were presented by “mean ± SD” and categorical data were presented by “frequency (%)”. ^†^Comorbidities were assessed within a year before the index date. ^††^SLE-related comorbidities were assessed during observational periods. ^§^Medication use for more than 30 days within a year including the index date.

**Supplementary Table 2. Comparison of the causes of death according to the time of death**

| Cause of death | Early death  (n=290) | Late death  (n=438) |
| --- | --- | --- |
|  | **No. of events (%)** | **No. of events (%)** |
| All cause | 290 (100.0) | 438 (100.0) |
| SLE related condition | 136 (46.9) | 84 (19.2) |
| Cardiovascular | 36 (12.4) | 81 (18.5) |
| Cancer | 29 (10.0) | 72 (16.4) |
| Infection | 34 (11.7) | 49 (11.2) |
| Renal | 9 (3.1) | 24 (5.5) |
| Rheumatoid | 10 (3.4) | 7 (1.6) |
| Respiratory | 5 (1.7) | 10 (2.3) |
| Liver | 4 (1.4) | 10 (2.3) |
| Haematologic | 2 (0.7) | 10 (2.3) |
| Sudden death | 3 (1.0) | 6 (1.4) |
| Gastrointestinal | 5 (1.7) | 4 (0.9) |
| Mental | 0 (0.0) | 8 (1.8) |
| Injury | 1 (0.3) | 4 (0.9) |
| Musculoskeletal | 1 (0.3) | 4 (0.9) |
| Accident/Suicide | 1 (0.3) | 2 (0.5) |
| Endocrine | 1 (0.3) | 2 (0.5) |
| Unknown | 7 (2.4) | 15 (3.4) |
| Uncategorised | 6 (2.1) | 46 (10.5) |

**Supplementary Table 3. Risk factors of early death among patients with SLE (n=11,375)**

| Variables | SLE-related death | | | |
| --- | --- | --- | --- | --- |
|  | **Univariable OR**  **(95% CI)** | **p-value** | **Multivariable OR**  **(95% CI)** | **p-value** |
| Age | 1.02 (1.01, 1.03) | 0.002 | 1.00 (0.99, 1.01) | 0.911 |
| Female | 0.75 (0.48, 1.17) | 0.199 | 0.96 (0.58, 1.57) | 0.857 |
| Medical aid | 0.17 (0.05, 0.54) | 0.003 | 0.07 (0.02, 0.24) | <0.001 |
| Comorbidities^†^ |  |  |  |  |
| Hypertension | 2.28 (1.60, 3.24) | <.0001 | 1.87 (1.15, 3.05) | 0.012 |
| Diabetes mellitus | 1.59 (0.83, 3.05) | 0.161 | 1.38 (0.64, 2.96) | 0.414 |
| Hyperlipidaemia | 0.94 (0.55, 1.61) | 0.823 | 0.40 (0.21, 0.79) | 0.008 |
| Chronic kidney disease | 1.70 (0.83, 3.49) | 0.150 | 0.69 (0.30, 1.60) | 0.391 |
| Cardiovascular disease | 3.28 (1.87, 5.76) | <.0001 | 1.57 (0.80, 3.11) | 0.193 |
| Cancer | 0.81 (0.33, 1.98) | 0.638 | 0.41 (0.15, 1.11) | 0.079 |
| SLE-related manifestations^††^ |  |  |  |  |
|  |  |  |  |  |
| Antiphospholipid antibody syndrome | 0.67 (0.21, 2.13) | 0.501 | 0.43 (0.12, 1.51) | 0.188 |
| Avascular necrosis | 0.67 (0.09, 4.83) | 0.691 | 0.84 (0.10, 6.97) | 0.870 |
| Interstitial lung disease | 4.08 (2.39, 6.94) | <.0001 | 2.87 (1.45, 5.70) | 0.003 |
| Pulmonary artery hypertension | 6.06 (2.60, 14.11) | <.0001 | 2.69 (0.94, 7.76) | 0.066 |
| Pulmonary alveolar haemorrhage | 20.60 (9.35, 45.37) | <.0001 | 2.21 (0.74, 6.60) | 0.154 |
| Lupus nephritis | 2.40 (1.69, 3.41) | <.0001 | 1.61 (1.06, 2.44) | 0.026 |
| SLE-related complications^††^ |  |  |  |  |
| Congestive heart failure | 5.31 (3.56, 7.93) | <.0001 | 3.12 (1.88, 5.17) | <0.001 |
| Opportunistic infection | 2.19 (1.37, 3.51) | 0.001 | 1.73 (1.01, 2.96) | 0.046 |
|  |  |  |  |  |
| Medication^§^ |  |  |  |  |
| Glucocorticoid (oral) | 0.27 (0.19, 0.38) | <.0001 | 0.19 (0.12, 0.31) | <0.001 |
| Glucocorticoid (intravenous) | 13.02 (6.83, 24.81) | <.0001 | 15.41 (7.85, 30.24) | <0.001 |
| Hydroxychloroquine | 0.21 (0.14, 0.30) | <.0001 | 0.29 (0.19, 0.45) | <0.001 |
| Nonsteroidal anti-inflammatory drug | 0.16 (0.09, 0.28) | <.0001 | 0.21 (0.12, 0.38) | <0.001 |
| Immunosuppressive agent (oral) | 0.34 (0.21, 0.55) | <.0001 | 0.22 (0.12, 0.40) | <0.001 |
| Cyclophosphamide (intravenous) | 5.35 (3.71, 7.71) | <.0001 | 4.66 (2.82, 7.70) | <0.001 |

OR, odds ratio; CI, confidence interval

^†^Comorbidities were assessed within a year before the index date. ^††^SLE-related manifestations and complications were assessed within a year including the index date. ^§^Medication use for more than 30 days within a year including the index date
